# Genomic Microevolution of *Vibrio cholerae* O1, Lake Tanganyika Basin, Africa

**DOI:** 10.3201/eid2901.220641

**Published:** 2023-01

**Authors:** Yaovi M.G. Hounmanou, Elisabeth Njamkepo, Jean Rauzier, Karin Gallandat, Aurélie Jeandron, Guyguy Kamwiziku, Klaudia Porten, Francisco Luquero, Aaron Aruna Abedi, Baron Bashige Rumedeka, Berthe Miwanda, Martin Michael, Placide Welo Okitayemba, Jaime Mufitini Saidi, Renaud Piarroux, François-Xavier Weill, Anders Dalsgaard, Marie-Laure Quilici

**Affiliations:** University of Copenhagen, Frederiksberg, Denmark (Y.M.G. Hounmanou, A. Dalsgaard);; Institut Pasteur, Université Paris Cité, Paris, France (E. Njamkepo, J. Rauzier, F.-X. Weill, M.-L. Quilici);; London School of Hygiene and Tropical Medicine, London, UK (K. Gallandat, A. Jeandron);; University of Kinshasa, Kinshasa, Democratic Republic of the Congo (G. Kamwiziku); Epicentre, Paris (K. Porten, F. Luquero);; Ministry of Public Health, Kinshasa (A. Aruna Abedi, P. Welo Okitayemba);; Ministry of Public Health, Uvira, Democratic Republic of the Congo (B. Bashige Rumedeka, J. Mufitini Saidi);; Institut National de Recherche Biomédicale, Kinshasa (B. Miwanda);; Sokoine University of Agriculture College of Veterinary Medicine and Biomedical Sciences, Morogoro, Tanzania (M. Michael);; Sorbonne Université, Inserm UMR 1136, Assistance Publique-Hôpitaux de Paris, Hôpital Pitié-Salpêtrière, Paris (R. Piarroux)

**Keywords:** cholera, *Vibrio cholerae*, bacteria, genomics, neglected tropical diseases, African Great Lakes Region, Lake Tanganyika, Tanzania, Democratic Republic of Congo, Burundi, Zambia, *Suggested citation for this article*: Hounmanou YMG, Njamkepo E, Rauzier J, Gallandat K, Jeandron A, Kamwiziku G, et al. Genomic microevolution of *Vibrio cholerae* O1, Lake Tanganyika Basin, Africa. Emerg Infect Dis. 2023 Jan [*date cited*]. https://doi.org/10.3201/eid2901.220641

## Abstract

Africa’s Lake Tanganyika basin is a cholera hotspot. During 2001–2020, *Vibrio cholerae* O1 isolates obtained from the Democratic Republic of the Congo side of the lake belonged to 2 of the 5 clades of the AFR10 sublineage. One clade became predominant after acquiring a *parC* mutation that decreased susceptibility to ciprofloxacin.

Cholera is an acute life-threatening diarrheal disease responsible for ≈4.3 million cases and 142,000 deaths annually worldwide (*1*). Excluding epidemic peaks in Haiti and Yemen (*2*,*3*), most cases of cholera originate from sub-Saharan Africa, predominantly the African Great Lakes Region (AGLR); specifically, the countries of the Lake Tanganyika basin (*4*). Many recurrent cholera outbreaks in the Democratic Republic of the Congo (DRC), Tanzania, Burundi, and Zambia have been linked to a common hotspot area around the Lake Tanganyika basin (*5*–*8*).

By the end of 2018, the World Health Organization had noted a steady decline in cholera cases throughout the world, including the AGLR (*9*). Continuous genomic surveillance of circulating *Vibrio cholerae* bacteria strains is required to understand the transmission dynamics and genetic evolution of *V. cholerae* and potentially to guide prevention and response interventions to continue the trend toward decreasing case numbers, in line with the global cholera roadmap to 2030 (*10*). One lineage, seventh pandemic *V. cholerae* O1 El Tor (7PET), is responsible for the current pandemic, which began in 1961 (*11*); Africa was hit by 7PET in 1970 (*11*). During 1970–2014, >11 different 7PET sublineages were introduced from South Asia into Africa, and sublineage AFR10 (previously T10) replaced AFR5 (previously T5) in the AGLR in the late 1990s (*11*). Sublineage AFR13 (previously T13) was identified in East Africa (Tanzania, Uganda, Kenya) and Zimbabwe (*12*). We tracked the 7PET populations circulating in the Lake Tanganyika basin by studying recent *V. cholerae* O1 isolates collected in the region by conventional bacteriology and genomics and placing these genomes in a broader phylogenetic context to elucidate their evolutionary history.

## The Study

**Figure 1:**
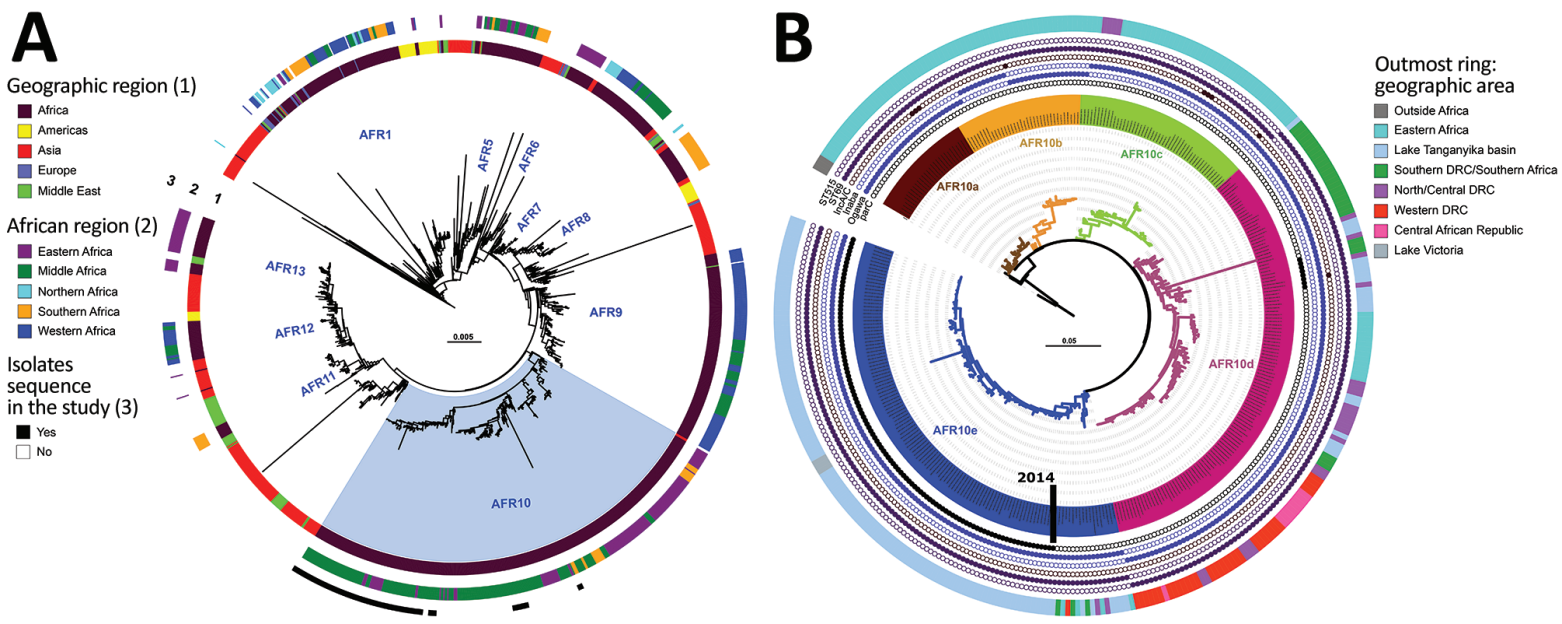
Phylogenomics of clinical and environmental *Vibrio cholerae* O1 El Tor isolates from the Lake Tanganyika basin, Africa. A) Maximum-likelihood phylogeny of 1,366 seventh pandemic *V. cholerae* O1 El Tor (7PET) genomes with strain A6 as the outgroup. The different sublineages introduced into Africa are indicated. Light blue indicates AFR10 sublineage. Rings 1 and 2 show geographic origin of isolates; ring 3 shows isolates sequenced in this study. B) Maximum-likelihood tree for 357 AFR10 isolates, with strain N16961 as an outgroup. The 5 clades are color-coded: AFR10a, brown; AFR10b, yellow; AFR10c, green; AFR10d, pink; and AFR10e, blue. The outermost ring indicates the geographic locations of the different isolates in the tree. Filled circles indicate the presence of ST69 or ST515, Ogawa and Inaba serotypes, IncA/C plasmid, and the S85L mutation in *par*C; open circles indicate their absence. MLST, multilocus sequence typing; ST, sequence type.

We analyzed 96 *V. cholerae* O1 isolates collected during 2015–2020 in DRC (86 clinical isolates, including 39 collected in 2018–2020) and Tanzania (10 environmental isolates from fish and lake water) (Appendix 1; Appendix 2 Table 1). We subjected the isolates to antimicrobial susceptibility testing, whole-genome sequencing, genomic characterization, and phylogenetic analyses, as previously described (*11*,*12*) (Appendix 1). We performed a phylogenetic analysis of these genomes within a global collection of 1,366 7PET *V. cholerae* O1 genomes (Appendix 2 Table 2), including another 130 genomes from DRC collected during 1984–2017. We based the final maximum-likelihood phylogenetic tree on 10,352 single-nucleotide variants distributed over the nonrepetitive, nonrecombinant core genome (Figure 1, panel A).

**Figure 2:**
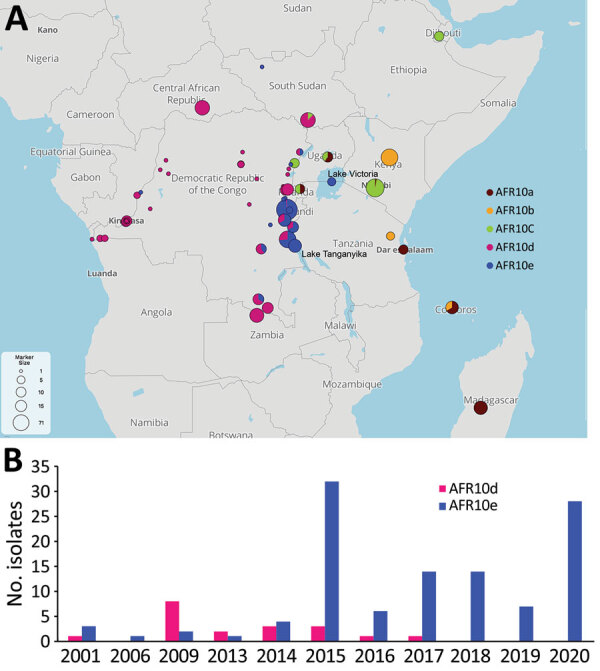
A) Spatiotemporal dynamics of the AFR10 clades of *Vibrio cholerae* O1 in the African Great Lakes Region, Africa, 1998–2020. Circle size indicates the number of isolates at the location concerned. The 5 AFR10 clades are color-coded: AFR10a, brown; AFR10b, yellow; AFR10c, green; AFR10d, pink; and AFR10e, blue. B) *V. cholerae* O1 isolates from the Lake Tanganyika basin. All AFR10 isolates from Bujumbura (Burundi), Kigoma (Tanzania) and the South-Kivu province (DRC) were considered to be Lake Tanganyika basin isolates.

Phylogenetic analysis of the 96 genomes of *V. cholerae* O1 isolates showed that all belonged to 7PET sublineage AFR10 (Figure 1, panel A). Within the limits of our sampling, sublineage AFR5, which circulated actively in the AGLR during the 1980s–1990s (*11*), appears to be extinct in the region, whereas sublineage AFR13, reported in 2015 in Uganda and Tanzania (*12*), has not yet spread to DRC. Since 1998, the endemicity of the AFR10 sublineage in the Tanganyika basin and surrounding countries has led to microevolution; an analysis of 357 AFR10 genomes from our global dataset revealed the presence of 5 clades, AFR10a–AFR10e (Figure 1, panel B; Appendix 1 Figure 1). Clades AFR10a, AFR10b, and AFR10c were mostly associated with the eastern AGLR countries. The isolates of these clades were of sequence type (ST) 69 (Figure 1, panel B). Clades AFR10d and AFR10e predominated in DRC and the Lake Tanganyika basin. Clade AFR10d is of ST515, essentially Inaba serotype, and was widespread in DRC and neighboring countries (Figure 2, panel A), as previously reported (*13*). It was the only clade found in the Lake Kivu and Lake Edward basins. AFR10e strains are of ST69, Ogawa serotype, and were essentially restricted to the Lake Tanganyika basin, confirming previous findings (*13*). A further pangenome analysis of the AFR10 isolates revealed no clade-specific gain or loss of genes (Appendix 1 Figure 2). AFR10e strains have gradually replaced AFR10d strains in the region since 2014; all *V. cholerae* O1 strains obtained from the Tanganyika basin by 2017, as well as those obtained from the lake itself in 2018 and 2019, were AFR10e strains (Figure 2 panel B). Epidemiologic studies identified cholera hotspots in the AGLR as a source of major countrywide outbreaks reaching the capital, Kinshasa, and the Atlantic coast, via the Congo River, in 2011, 2012, and 2016 (*5*). These outbreaks were caused primarily by clade AFR10d, which has a wider geographic distribution than clade AFR10e (Figure 2) (*14*).

One striking characteristic of AFR10e isolates was the presence of a mutation in the quinolone resistance–determining region of the topoisomerase IV subunit A gene, *parC* (S85L), that led to higher MIC values (0.25–0.38 mg/L) for ciprofloxacin. These isolates, which have a lower susceptibility to ciprofloxacin than wild-type populations, would be classified as either resistant (MIC 0.38 mg/L) or susceptible (MIC 0.25 mg/L) in accordance with clinical breakpoints published by the European Committee on Antimicrobial Susceptibility Testing (https://www.eucast.org/clinical_breakpoints) in 2022, as inferred from the study of *Vibrio* strains and not Enterobacteriaceae strains. This *parC* mutation, sporadically reported in other AFR10 clades (Table; Figure 1, panel B; Appendix 2 Table 1), has been a distinctive characteristic of AFR10e isolates since 2014 (Figure 2, panel B). It was the second mutation affecting susceptibility to quinolones and fluroquinolones to be found in this AFR10e clade; the first was a mutation in the DNA gyrase subunit A gene, *gyrA* (S83I), present in all AFR10 isolates. This additional mutation does not seem to be associated with the specific use of fluoroquinolones for treating cholera outbreaks, because the antimicrobial drugs used for first-line cholera control in DRC are tetracyclines and macrolides, to which AFR10e isolates remain susceptible. Instead, the mutation may result from widespread self-medication with antimicrobials, a common practice in many sub-Saharan Africa countries including DRC (*15*).

**Table:** Characteristics of *Vibrio cholerae* O1 isolates from humans and the environment around Lake Tanganyika, Africa, 2015–2020*

Characteristic	2015	2016	2017	2018	2019	2020
No. isolates	26	7	14	14	7	28
AFR10 clade	AFR10d (1)	AFR10d (5)	AFR10e	AFR10e	AFR10e	AFR10e
	AFR10e (25)	AFR10e (2)				
Serotype	Inaba (1)	Inaba (5)	Ogawa	Ogawa	Ogawa	Ogawa
	Ogawa (25)	Ogawa (2)				
Source	Human	Human	Human	Human (10)	Human (1)	Human
				Fish (2)	Fish (4)	
				Water (2)	Water (2)	
MLST	ST515 (1)	ST515 (5)	ST69	ST69	ST69	ST69
	ST69 (25)	ST69 (2)				
Antimicrobial resistance determinants						
SXT/R391 element	ICE*Vch*Ind5	ICE*Vch*Ind5	ICE*Vch*Ind5†	ICE*Vch*Ind5	ICE*Vch*Ind5	ICE*Vch*Ind5
* gyrA*	S83I	S83I	S83I	S83I	S83I	S83I
* parC*	S85L	S85L (2), WT (5)	S85L	S85L	S85L	S85L
VC_0715	R169C	R169C	R169C	R169C	R169C	R169C
VC_A0637	Q5Stop	Q5Stop	Q5Stop	Q5Stop	Q5Stop	Q5Stop
AMR	AMR1‡ (26)	AMR1‡ (2)	AMR1‡ (13)	AMR1 (14)‡	AMR1 (7)‡	AMR1 (28)‡
		AMR2§ (5)	AMR3¶ (1)			

*Numbers in parentheses indicate number of isolates for each designation. AMR, antimicrobial resistance patterns; CIP, ciprofloxacin; DS, decreased susceptibility; FUR, nitrofurantoin; MLST, multilocus sequence typing; NAL, nalidixic acid; O129, cross-resistance to the vibriostatic agent O/129 and trimethoprim; PMB, polymyxin B; SSS, sulfonamides; ST, sequence type; STR, streptomycin; SXT, trimethoprim/sulfamethoxazole †Deletion of the *strA*, *strB*, *floR*, and *sul2* genes in isolate CNRVC170308 (all isolates with intact ICE*Vch*Ind5 harbor the *strA*, *strB*, *dfrA1*, *floR*, and *sul2* resistance genes) ‡Resistance to STR, SSS, O129, SXT, FUR, PMB, NAL, and CIP^DS^. §Resistance to STR, SSS, O129, SXT, FUR, PMB, and NAL. ¶Resistance to O129, FUR, PMB, NAL, CIP^DS^.

All 96 isolates analyzed had known mutations of the *VC_0715* and *VC_A0637* genes conferring nitrofuran resistance (Table), consistent with previous findings (*2*). The isolates also carried the SXT/R391 genomic element ICE*Vch*Ind5, encoding resistance to streptomycin (*strAB*), sulfonamides (*sul2*), chloramphenicol (*flo*R), trimethoprim and the O/129 vibriostatic agent (*dfrA1*), and trimethoprim–sulfamethoxazole (*sul2* and *dfrA1*), with concordance between the phenotypic and genotypic data (Table; Appendix 2 Table 1).

## Conclusions

We found that the cholera outbreaks in the eastern part of DRC during 2001–2020 were caused by *V. cholerae* O1 sublineage AFR10, which was introduced into East Africa from South Asia in the late 1990s. The AFR13 sublineage was already reported in 2015 in Tanzania, including the city of Kigoma, was located on the shore of Lake Tanganyika but had not been detected in DRC as of 2022. The AFR10 isolates of this region belong principally to 2 clades, AFR10d (Inaba, ST515) and AFR10e (Ogawa, ST69). AFR10d was responsible for outbreaks reported in the western part of DRC in 2011–2017 and neighboring countries; AFR10e (Ogawa, ST69) was restricted to the Lake Tanganyika basin, in which reduced susceptibility to ciprofloxacin has been seen since 2014. Lake Tanganyika seems to serve as a transmission channel, favoring the establishment of AFR10e in local human populations. Further investigation, including studies of population movement, should reveal why AFR10e clade has remained within the Lake Tanganyika basin. The replacement of other clades by this antimicrobial-resistant clade in this area highlights the need for more systematic documentation of antimicrobial drug use and the implementation of adapted stewardship programs, particularly in outbreak responses. Overall, these findings highlight the need for continuous genomic surveillance and for coordinated communication between countries for effective interventions.

Appendix 1 Additional information about the methodology used in study of genomic microevolution of *Vibrio cholerae* O1 in the Lake Tanganyika basin.

Appendix 2 Genomic data and metadata for the genomes analyzed in study of microevolution of *Vibrio cholerae* O1 in the Lake Tanganyika basin.
