# Supplementary material for: Genomic Microevolution of Vibrio cholerae O1, Lake Tanganyika Basin, Africa
Source: Emerg Infect Dis. 2023 Jan;29(1):149–53. doi: 10.3201/eid2901.220641 (PMC9796204; doi:10.3201/eid2901.220641)
Supplement: Appendix 1 — Additional information about the methodology used in study of genomic microevolution of Vibrio cholerae O1 in the Lake Tanganyika basin. [file 22-0641-Techapp-s1.pdf]

Article DOI: https://doi.org/10.3201/eid2901.220641

Genomic Microevolution of *Vibrio cholerae* O1, Lake Tanganyika Basin, Africa

Appendix 1

Detailed Materials and Methods

Environmental Isolates from Lake Tanganyika

Fieldwork was conducted during September 2018–March 2019, a period including a dry and a rainy season, at 2 sites on the Tanzania side of Lake Tanganyika: Kibirizi, 2.2 km north of Kigoma, and Katonga, 4–5 km south of Kigoma Bay. Samples were collected after the major cholera outbreak that occurred in 2015–2018 in all 26 regions of the United Republic of Tanzania (https://www.who.int/emergencies/disease-outbreak-news/item/12-january-2018-cholera-tanzania-en). Since April 2018, the number of reported cases has fallen considerably in Kigoma, but cholera cases were still being reported right up until the end of 2018 (https://reliefweb.int/report/zimbabwe/bulletin-cholera-and-awd-outbreaks-eastern-and-southern-africa-regional-update-5) and early 2019 (https://reliefweb.int/report/mozambique/bulletin-cholera-and-awd-outbreaks-eastern-and-southern-africa-regional-update).

In total, 140 individual fish (*Oreochromis tanganicae*), each weighing ≈300 g and captured from deep offshore waters, were purchased directly from fishing boats, as soon as they landed at the 2 landing sites at Kibirizi and Katonga. During sample processing, each fish was subdivided into 2 subsamples corresponding to the gills and intestines, yielding a total of 280 subsamples for analysis.

We also collected 60 water samples (2 L each) over the sampling period. Each sample was collected ≥500 m from the shore, at the Kibirizi and Katonga landing sites, at a depth of ≈1 m from the surface. The samples were analyzed according to the protocols described in our previous articles, including an initial enrichment step in alkaline peptone water (*1*,*2*). The identity of the bacterial isolates was confirmed by PCR targeting the *V. cholerae*–specific outer membrane protein gene *ompW* and the cholera enterotoxin *ctxA* gene. The samples were then

serotyped with polyvalent O1 antiserum. Ten of the *V. cholerae* isolates recovered were confirmed to be *ctxA*-positive *V. cholerae* O1: 6 from fish (gills) and 4 from water. These 10 isolates were shipped to the University of Copenhagen, where they were tested for antimicrobial resistance patterns as described below. DNA was extracted with a Maxwell automatic purification machine and kit (Promega, https://www.promega.com) according to the manufacturer's protocol and were fully sequenced with the Illumina MiSeq platform (*2*,*3*), with 40× to 75× coverage. The paired-end reads obtained were submitted to the European Nucleotide Archive under accession number PRJEB37131.

Clinical *V. cholerae* O1 Isolates from the Democratic Republic of Congo (DRC)

Isolates collected in 2015 and 2016 (n = 17) from Uvira, Kinshasa, Lubumbashi, and Bunia were provided by Kinshasa University. Stool samples were collected by Médecins Sans Frontières France at the Kalemie Cholera Treatment Center (CTC) during October 2015–February 2016, as part of the cholera surveillance program, following the use of a bivalent oral cholera vaccine in the city of Kalemie. Samples from Uvira were collected during 2017–2020 in the context of a study conducted by the London School of Hygiene and Tropical Medicine (LSHTM) with the aim of evaluating the impact of urban water supply improvements on cholera and other diarrheal diseases in Uvira, DRC (www.clinicaltrials.gov identifier: NCT02928341, ethics approval from the LSHTM 8913, 10603 and from the Ethics Committee from the School of Public Health, University of Kinshasa, DRC ESP/CE/088/2015). Rectal swabs from patients with severe acute diarrhea admitted to the CTC at Uvira General Hospital were subjected to a six-hour enrichment procedure in alkaline peptone water (APW) at room temperature and were tested with a rapid diagnostic test (Crystal VC, Span Diagnostics, https://span-diagnostics.com). Rectal swab samples subjected to enrichment in APW that tested positive with cholera rapid diagnostic tests were preserved on a 6 mm strip of filter paper in a capped tube containing a few drops of saline ("wet filter sample"), and were transferred, in batches, from Uvira to Institut Pasteur at room temperature, in accordance with IATA transport restrictions for category B infectious substances.

Bacterial isolates, stool samples and enriched samples were sent to the Centre National de Référence des Vibrions et du Cholera (CNRVC), Institut Pasteur, Paris, France, for the confirmation of cholera diagnosis by culture according to standard protocols (*4*) and/or PCR (*5*)

and for further characterization to support epidemiologic surveillance; 16 isolates from stools (Kalemie) and 53 from enriched samples (Uvira) were recovered and added to the study.

Total DNA was extracted from the clinical isolates with the Maxwell 16-cell DNA purification kit (Promega, https://www.promega.com) in accordance with the manufacturer's instructions. Whole-genome sequencing was carried out on the Biomics and PIBnet sequencing platforms of the Institut Pasteur and at the *Institut du Cerveau* platform (Paris), on Illumina NextSeq or Novaseq platforms (Illumina, https://www.illumina.com) generating 100–250 bp paired-end reads, yielding 110x to 728x coverage. All the publicly available genomes added to the analysis are referenced in Appendix 2 Table 2. Short reads from the clinical isolates were submitted to ENA under accession number PRJEB47296.

Antimicrobial Susceptibility Testing

All the *V. cholerae* isolates analyzed in this study were subjected to antimicrobial susceptibility testing at the University of Copenhagen (environmental isolates) and the CNRVC, Institut Pasteur, Paris, France (clinical isolates). Antimicrobial susceptibility testing was performed by the disk diffusion method, on Mueller-Hinton agar (Bio-Rad, https://www.bio-rad.com), in accordance with EUCAST guidelines (https://www.eucast.org). The antimicrobial drugs tested were: ampicillin, cefalotin, cefotaxime, streptomycin, chloramphenicol, azithromycin, sulfonamides, trimethoprim-sulfamethoxazole, vibriostatic agent O/129, tetracycline, nalidixic acid, ciprofloxacin, nitrofurantoin, and polymyxin B. *Escherichia coli* CIP76.24 (ATCC25922) was used as a control. The MICs of nalidixic acid and ciprofloxacin were determined with Etests (bioMérieux, https://www.biomerieux.com). Interpretation of the MIC results for ciprofloxacin was based on the EUCAST 2022 clinical breakpoints for *Vibrio* species (https://www.eucast.org/clinical_breakpoints).

Genome Analysis

Trimmed reads were assembled with SPAdes version 3.15.2 (*6*). *In silico* multilocus sequence typing (MLST) for *V. cholerae* was performed with PubMLST (https://pubmlst.org/bigsdb?db = pubmlst_vcholerae_seqdef). Specific genetic markers for *V. cholerae* were analyzed with BLAST version 2.2.26. against reference sequences of the *rfb* O1gene, *ctxB*, *wbeT*, and the whole VSP-II locus, as previously described (*7*). Acquired antimicrobial resistance genes were predicted with ResFinder version 4.0.1

(https://cge.food.dtu.dk/services/ResFinder), BLAST analysis against GI-15, Tn*7*, and SXT/R391 integrative and conjugative elements, and PlasmidFinder version 2.1.1. (https://cge.food.dtu.dk/services/PlasmidFinder). The presence of mutations in genes associated with resistance to quinolones (*gyrA*, *gyrB, parC, parE*) and nitrofurans (*VC_0715* and *VC_A0637*) was investigated with in-house tools, using BLAST version 2.2.26, as previously described (*8*).

Phylogenetic Analysis

Single-nucleotide variants (SNVs) were called with Snippy version 4.6.0 /Freebayes version 1.3.2 (https://github.com/tseemann/snippy) under the following constraints: mapping quality of 60, a minimum base quality of 13, a minimum read coverage of 4, and 75% read concordance at a locus for a variant to be reported relative to the *V. cholerae* O1 El Tor N16961 reference genome. An alignment of core genome SNVs was produced in Snippy version 4.1.0 for phylogeny inference. Repetitive (insertion sequences and the TLC-RS1-CTX region) and recombinogenic (VSP-II) regions in the alignment were masked (*9*). Putative recombinogenic regions were detected and masked with Gubbins version 2.4.1 (*10*). A maximum likelihood (ML) phylogenetic tree was built, with RAxML version 8.2.12, under the GTR model, with 200 bootstraps (*11*). The final tree was rooted on the A6 genome and visualized with iTOL v5 (*12*) or FigTree version 1.4.2. (http://tree.bio.ed.ac.uk/software/figtree).

Pangenome Analysis

We annotated 355 of the 357 genomes of the AFR10 sublineage (the assemblies for the Tanz_100 and Tanz_20 strains being excluded due to light contamination with non-*Vibrio* species sequences) with Prokka version 1.13.4 (*13*). The annotated GFF3 files were then used as an input for the Roary (version.3.7.0) (*14*) pangenome analysis tool in a Linux interface. We used the binary presence/absence data of the accessory genome produced in Roary, together with the tree of AFR10 strains, for visualization in Phandango (*15*). The results are shown in Appendix 1 Figure 2.

References

1. Hounmanou YMG, Mdegela RH, Dougnon TV, Mhongole OJ, Mayila ES, Malakalinga J, et al. Toxigenic *Vibrio cholerae* O1 in vegetables and fish raised in wastewater irrigated fields and stabilization ponds during a non-cholera outbreak period in Morogoro, Tanzania: an environmental health study. BMC Res Notes. 2016;9:466. PubMed https://doi.org/10.1186/s13104-016-2283-0

2. Hounmanou YMG, Leekitcharoenphon P, Hendriksen RS, Dougnon TV, Mdegela RH, Olsen JE, et al. Surveillance and genomics of toxigenic *Vibrio cholerae* O1 from fish, phytoplankton and water in Lake Victoria, Tanzania. Front Microbiol. 2019;10: 901. https://www.frontiersin.org/articles/10.3389/fmicb.2019.00901/full

3. Hounmanou YMG, Leekitcharoenphon P, Kudirkiene E, Mdegela RH, Hendriksen RS, Olsen JE, et al. Genomic insights into *Vibrio cholerae* O1 responsible for cholera epidemics in Tanzania between 1993 and 2017. PLoS Negl Trop Dis. 2019;13:e0007934. PubMed https://doi.org/10.1371/journal.pntd.0007934

4. Dodin A, Fournier JM. Diagnosis of the cholera vibrio. Laboratory methods for the diagnosis of cholera vibrio and other vibrios. Institut Pasteur Paris (France); 1992. p. 59–82.

5. Fields PI, Popovic T, Wachsmuth K, Olsvik O. Use of polymerase chain reaction for detection of toxigenic *Vibrio cholerae* O1 strains from the Latin American cholera epidemic. J Clin Microbiol. 1992;30:2118–21. PubMed https://doi.org/10.1128/jcm.30.8.2118-2121.1992

6. Bankevich A, Nurk S, Antipov D, Gurevich AA, Dvorkin M, Kulikov AS, et al. SPAdes: a new genome assembly algorithm and its applications to single-cell sequencing. J Comput Biol. 2012;19:455–77. PubMed https://doi.org/10.1089/cmb.2012.0021

7. Breurec S, Franck T, Njamkepo E, Mbecko JR, Rauzier J, Sanke-Waïgana H, et al. Seventh pandemic *Vibrio cholerae* O1 sublineages, Central African Republic. Emerg Infect Dis. 2021;27:262–6. PubMed https://doi.org/10.3201/eid2701.200375

8. Weill FX, Domman D, Njamkepo E, Almesbahi AA, Naji M, Nasher SS, et al. Genomic insights into the 2016–2017 cholera epidemic in Yemen. Nature. 2019;565:230–3. PubMed https://doi.org/10.1038/s41586-018-0818-3

9. Weill FX, Domman D, Njamkepo E, Tarr C, Rauzier J, Fawal N, et al. Genomic history of the seventh pandemic of cholera in Africa. Science. 2017;358:785–9. PubMed https://doi.org/10.1126/science.aad5901

10. Croucher NJ, Page AJ, Connor TR, Delaney AJ, Keane JA, Bentley SD, et al. Rapid phylogenetic analysis of large samples of recombinant bacterial whole genome sequences using Gubbins. Nucleic Acids Res. 2015;43:e15–15. PubMed https://doi.org/10.1093/nar/gku1196

11. Stamatakis A. RAxML version 8: a tool for phylogenetic analysis and post-analysis of large phylogenies. Bioinformatics. 2014;30:1312–3. PubMed https://doi.org/10.1093/bioinformatics/btu033

12. Letunic I, Bork P. Interactive tree of life (iTOL) v3: an online tool for the display and annotation of phylogenetic and other trees. Nucleic Acids Res. 2016;44:W242–245. https://doi.org/10.1093/nar/gkw290

13. Seemann T. Prokka: rapid prokaryotic genome annotation. Bioinformatics. 2014;30:2068–9. PubMed https://doi.org/10.1093/bioinformatics/btu153

14. Page AJ, Cummins CA, Hunt M, Wong VK, Reuter S, Holden MTG, et al. Roary: rapid large-scale prokaryote pan genome analysis. Bioinformatics. 2015;31:3691–3. PubMed https://doi.org/10.1093/bioinformatics/btv421

15. Hadfield J, Croucher NJ, Goater RJ, Abudahab K, Aanensen DM, Harris SR. Phandango: an interactive viewer for bacterial population genomics. Bioinformatics. 2018;34:292–3. PubMed https://doi.org/10.1093/bioinformatics/btx610

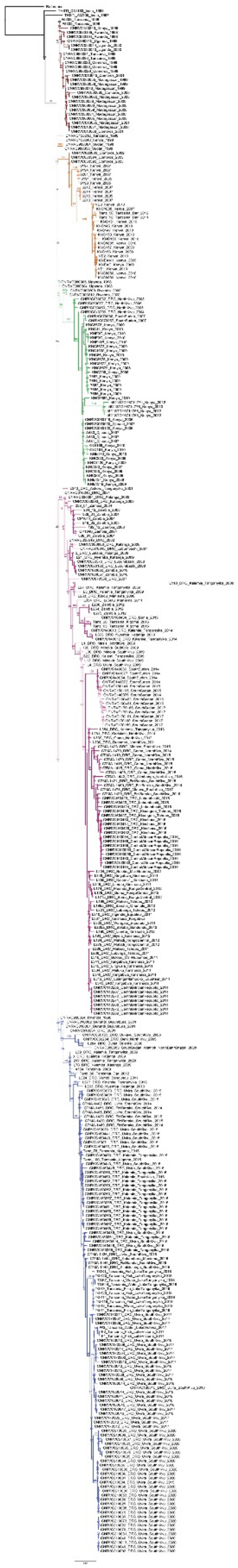

Appendix Figure 1. Detailed phylogenetic tree for the AFR10 sublineage isolates with bootstrap values at nodes. Branches are color-coded: AFR10a, brown; AFR10b, yellow; AFR10c, green; AFR10d, pink; and AFR10e, blue.

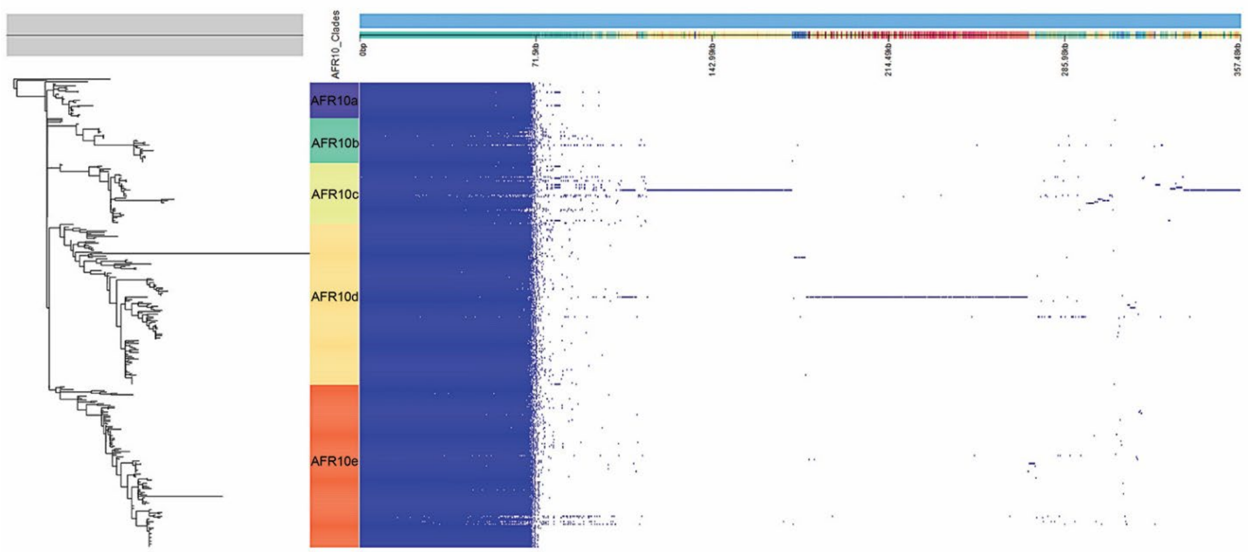

Appendix Figure 2. Pangenome analysis of 355 AFR10 isolates, using Roary version 3.7.0. On the left, the maximum-likelihood tree as in Appendix Figure 1, except for the 2 contaminated genomes, which were excluded. The 5 clades, AFR10a, AFR10b, AFR10c, AFR10d, and AFR10e, are color coded. On the right, pangenomic matrix, sorted from core genes on the left to accessory genes on the right, shows the absence (white) and presence (blue) of genes. The multicolor line (top) indicates the size of contigs in kilobases (kb).
